# Compensating control participants when the intervention is of significant value: experience in Guatemala, India, Peru and Rwanda

**DOI:** 10.1136/bmjgh-2019-001567

**Published:** 2019-08-21

**Authors:** Ashlinn K Quinn, Kendra Williams, Lisa M Thompson, Ghislaine Rosa, Anaité Díaz-Artiga, Gurusamy Thangavel, Kalpana Balakrishnan, J Jaime Miranda, Joshua P Rosenthal, Thomas F Clasen, Steven A Harvey

**Affiliations:** 1 Fogarty International Center, National Institutes of Health, Bethesda, Maryland, USA; 2 Division of Pulmonary and Critical Care Medicine, Johns Hopkins University School of Medicine, Baltimore, Maryland, USA; 3 Nell Hodgson Woodruff School of Nursing, Emory University, Atlanta, Georgia, USA; 4 Department of Disease Control, Faculty of Infectious and Tropical Diseases, London School of Hygiene and Tropical Medicine, London, UK; 5 Centro de Estudios en Salud, Universidad del Valle de Guatemala, Guatemala City, Guatemala; 6 Department of Environmental Health Engineering, Sri Ramachandra Insitute of Higher Education and Research, Chennai, Tamil Nadu, India; 7 CRONICAS Center of Excellence in Chronic Diseases and Department of Medicine, School of Medicine, Universidad Peruana Cayetano Heredia, Lima, Peru; 8 Department of Environmental Health, Rollins School of Public Health, Emory University, Atlanta, Georgia, USA; 9 Department of International Health, Johns Hopkins Bloomberg School of Public Health, Johns Hopkins University, Baltimore, Maryland, USA

**Keywords:** randomised controlled trial, compensation, ethics, multi-country trial

## Abstract

The Household Air Pollution Intervention Network (HAPIN) trial is a randomised controlled trial in Guatemala, India, Peru and Rwanda to assess the health impact of a clean cooking intervention in households using solid biomass for cooking. The HAPIN intervention—a liquefied petroleum gas (LPG) stove and 18-month supply of LPG—has significant value in these communities, irrespective of potential health benefits. For control households, it was necessary to develop a compensation strategy that would be comparable across four settings and would address concerns about differential loss to follow-up, fairness and potential effects on household economics. Each site developed slightly different, contextually appropriate compensation packages by combining a set of uniform principles with local community input. In Guatemala, control compensation consists of coupons equivalent to the LPG stove’s value that can be redeemed for the participant’s choice of household items, which could include an LPG stove. In Peru, control households receive several small items during the trial, plus the intervention stove and 1 month of fuel at the trial’s conclusion. Rwandan participants are given small items during the trial and a choice of a solar kit, LPG stove and four fuel refills, or cash equivalent at the end. India is the only setting in which control participants receive the intervention (LPG stove and 18 months of fuel) at the trial’s end while also being compensated for their time during the trial, in accordance with local ethics committee requirements. The approaches presented here could inform compensation strategy development in future multi-country trials.

**Summary box**

- Compensation packages were developed for households randomised to the control arm of an interventional trial in Guatemala, India, Peru and Rwanda, with the aim of reducing differential bias and incentivising continued engagement.
- A two-step process involving development of guiding principles followed by site-specific formative research was undertaken.
- The resultant compensation packages differed across the four sites while adhering to the overall guiding principles.
- The adaptive, context-specific approach taken here could inform control compensation in future multi-country trials.

## Introduction

The Household Air Pollution Intervention Network trial (HAPIN) is a multi-country randomised controlled trial (RCT) of a clean cookstove and fuel intervention for pregnant women who cook with biomass fuels, such as wood and dung. The trial seeks to evaluate the effect of switching to clean fuel cooking during pregnancy and the infant’s first year of life on the primary outcomes of birth weight, linear growth and severe pneumonia in the child, as well as blood pressure in older adult women who reside with the pregnant women. In total, 3200 pregnant women are being enrolled, 800 each in Guatemala, India, Peru and Rwanda. Activities are led by country-specific Intervention Research Centres (IRCs). Households randomised to the intervention arm receive a liquefied petroleum gas (LPG) stove and a continuous supply of free LPG from early pregnancy through the child’s first birthday, approximately 18 months per participant. The issue of appropriate compensation for households randomised to the control arm of HAPIN was the subject of extensive discussions and data gathering during the HAPIN formative research period, reported here.

Compensation packages for participation in research vary widely, including in low-income and middle-income country settings.^1^ Much of the literature has concerned appropriate levels of financial compensation, reflecting the fact that cash is a typical form of compensation in the USA and Europe.^2^ Cash is often valued over in-kind goods because it provides maximal flexibility in how it is spent or saved. Discussions of goods-based and service-based compensation are more rare, despite the fact that these types of compensation are often provided in low-income and middle-income country settings.^3–5^

In randomised trials, control compensation frequently consists of deferred receipt of the intervention. HAPIN initially conceived of control compensation similarly, planning to provide control participants with a stove and 18 months of LPG at the conclusion of trial participation. In early discussions, however, this approach was deemed inadequate for HAPIN for three primary reasons.

First, HAPIN investigators worried that asking control households to wait 18 months before receiving compensation might introduce differential bias into the trial. Participation in HAPIN requires a substantial time commitment despite the fact that ethical reviews concluded that the risks of participation in HAPIN were minimal. HAPIN investigators reasoned that the free, continuous supply of cooking fuel provided to intervention households would serve as sufficient incentivisation for intervention participants to stay enrolled for the 18 months of follow-up. Control participants would not receive this ongoing benefit, and for this reason might elect not to participate in the trial (potentially leading to selection bias), or might drop out of the trial at higher rates (resulting in differential losses to follow-up between arms).

Second, it is possible that the intervention could affect health outcomes by virtue of household economics rather than via a reduction in household air pollution. Intervention households would not need to purchase or collect their own biomass fuel for the duration of the trial. Thus, the time and money previously spent on fuel could theoretically be spent in ways that could impact trial outcomes such as birth weight and child growth, for example, by diverting money that would have been spent on fuel to more nutritious food, or using time saved from collecting fuel to visit the health centre more frequently.

Lastly, HAPIN investigators were concerned about the perceptions of fairness at the community level that could emerge as a result of asking control households to participate in a trial over an 18-month period without receiving any ongoing compensation while intervention households in the same communities were receiving a valuable asset (LPG stove and free fuel) immediately. Participation in HAPIN involves visits to health clinics for ultrasound examinations and other assessments, and numerous home visits for environmental sampling, child assessments of pneumonia and anthropometry, and collection of blood spot and urine for biomarkers. The HAPIN team worried that without ongoing compensation to control households there could be resentment between households that could create social disharmony and/or make the trial unwelcome in the community.

As the team began formative research, it became clear that the original plan of deferred receipt of the intervention could create logistical, scientific and potentially ethical problems. Designing appropriate incentives for control households therefore became of paramount importance. The compensation strategy needed to achieve the same goals in four diverse settings while also being contextually appropriate and satisfying local ethics authorities.

The work described here was approved by ethics review entities at Emory University, Johns Hopkins University, Universidad del Valle de Guatemala, the Guatemalan Ministry of Health, Sri Ramachandra Institute of Higher Education and Research, the Indian Council of Medical Research, the A.B. PRISMA, the London School of Tropical Hygiene and Medicine, and the Rwandan National Ethics Committee. HAPIN is registered at clinicaltrials.gov under identifier NCT02944682.

Development of control compensation packages occurred in two phases. First, a set of multi-country principles was developed for the overall trial. Next, each IRC conducted formative research to build a package consistent with those principles and acceptable to study participants, trial investigators and ethics committees. HAPIN is currently tracking the effectiveness of these strategies at achieving the objectives mentioned earlier. This paper describes the compensation packages identified and the considerations informing their development. Outcomes will be analysed and presented after the trial concludes in 2021.

## Guiding principles

The principles underlying HAPIN’s control compensation strategy were developed in discussions with bioethicists and ethics committees, and are shown in table 1. In short, they expressed three aims: (i) treat participants ethically, (ii) encourage controls to remain enrolled throughout the study and (iii) minimise the likelihood that the provision of LPG stoves and fuel to intervention households could create economic differences that might affect the health outcomes of the trial.

**Table 1:** Multi-country guidance for development of compensation strategies

**Aims**	Control households will receive compensation designed to meet three aims: (i) comply with applicable ethics requirements for treatment of controls, (ii) compensate control participants for the burden associated with the study, with a view to minimising losses to follow-up and (iii) offset the likely/potential economic advantage to intervention households accorded by the provision of free stoves and fuel
**Trial-wide principles**	1. Control compensation should not have the potential for impacting the outcomes of interest. For example, directly providing food would not be appropriate since it could impact HAPIN outcomes such as child growth and birth weight
	2. Any strategy to be incorporated into the trial cannot intentionally incentivise practices or behaviours that might put the control group at risk of adverse health outcomes. For example, provision of wood fuel would not be appropriate. On the other hand, incentives can be conditioned on continued participation in the trial
	3. The strategy should be created in consideration of the potential for other adverse effects of the compensation, such as disputes about who in the household receives and can use the incentive
	4. The strategies must be approved by applicable ethics committees and the HAPIN Data and Safety Monitoring Board

HAPIN, Household Air Pollution Intervention Network.

## Setting-specific compensation plans

With this guidance, the IRCs designed formative research activities to further develop setting-specific compensation plans. Table 2 outlines each country’s activities. Each IRC determined the contextually appropriate combination of formative research activities for their setting, according to their prior experience conducting similar activities in the area. Participants were generally drawn from pools of individuals who would be similar to HAPIN participants (eg, pregnant women or mothers with children under one from the community, their spouses and family members, and/or community members who had participated in a pilot study involving an LPG stove who would have familiarity with this technology and the burden of participating in a research study). The IRCs were in contact with one another throughout the design and conduct of the formative research through weekly team meetings held via teleconference. The progress and results of these activities were also discussed during an in-person meeting in September 2017.

**Table 2:** Formative research activities at each Intervention Research Centre

Country	Activity	Participants	Example of task
Guatemala	Working groups	Four working groups with 52 participants total: women and their spouses and/or other adult household members, all of whom had participated in a pilot of the LPG stove intervention	Asked to list household items a control household might like to have (within constraints); top responses voted on
India	In-depth interviews	20 participants, with purposive mix of (1) participants in an LPG pilot, (2) participants considered for the pilot who did not meet inclusion criteria and (3) new participants with no previous study connexion	Semi-structured interview on perspectives about randomised controlled trial participation, feelings about selection as a control and possible compensation for control participants
	Formal discussion	Six village health nurses in study area	Same semi-structured interview as for in-depth interviews (see above)
Peru	In-depth interviews	Seven pregnant women	Asked about typical gifts received by new mothers, typical fuel collection/purchasing practices and perceptions about LPG
	Focus group	One discussion with seven women (either pregnant or new mothers)	Discussed common household expenditures, valued items for women and new mothers, and methods for showing appreciation to those who provide help
Rwanda	Survey in antenatal clinics	60 pregnant women	Questions covered acceptability of different compensation strategies, including types of compensation, frequency of compensation and waiting periods for larger items
	Focus group discussions	Two discussions, each with eight former participants in LPG pilot and their husbands	Discussion of options to maintain control participants’ engagement and to ensure participants maintain trust that the study team will deliver larger items at the end of the trial

LPG, liquefied petroleum gas.

Lastly, each HAPIN IRC used the information gathered from the formative research activities to propose compensation packages for HAPIN participants. These were subsequently presented to the HAPIN investigators, institutional ethics committees and the HAPIN Data Safety Monitoring Board for approval. In some cases, feedback from these groups was incorporated into the final packages, with major changes discussed below.

The final compensation packages for HAPIN households are described in table 3. The specifics of the compensation plans differ by IRC in terms of timing of delivery, the nature of the items provided and the total cost per household.

**Table 3:** Planned compensation for control households

	Peru	Guatemala	India	Rwanda
Eligibility visit	--	--	Transportation/food allowance (INR* 200/US$3) for all potential participants†	--
Consent	--	--	Mock randomisation lunchbox worth INR 50 (<US$1) for all consented participants†	--
Baseline visit	--	--	Time compensation (INR 150/US$2) and transportation/food allowance (INR 200/US$3)†	Table (RWF 8000/US$10)
24–32 weeks of gestation	New hat for pregnant woman; New hat for older woman (PEN* 15/US$5)	Coupon worth GTQ* 500/US$67, to be saved or redeemed for items from a catalogue	Time compensation ×2 (INR 300/US$4) and transportation food allowance (INR 200/US$3)†	Kitenge cloth for pregnant woman and older woman (RWF 7000/US$8, each)
First post-birth visit	Gift bag with diaper bag, baby clothes, baby blanket (PEN 40/US$12)	Coupon worth GTQ 500/US$67, to be saved or redeemed for items from a catalogue	Newborn gift box with baby clothes, baby towel (INR 200/US$3)† Time compensation (INR 150/US$2)†	Gift bag with baby towel (1) baby clothes (three items), baby blanket (RWF 25 000/US$30)
3-month post-birth visit	--	--	Time compensation (INR 150/US$2)†	Gift bag with baby sheet (1) and baby clothes (three items) (RWF 15 500/US$19)
6-month post-birth visit	New sweater for mother (PEN 20/US$6) New sweater for adult woman (PEN 20/US$6) Child hat, toy (PEN 10/US$3)	Coupon worth GTQ 500/US$67, to be saved or redeemed for items from a catalogue	Time compensation (INR 150/US$2)†	Gift bag with baby clothes (2) and weaning utensils (RWF 13 900/US$17)
9-month post-birth visit	--	--	Time compensation (INR 150/US$2)†	Gift bag with baby clothes (1) baby shoes (2) toothpaste (2) and toothbrush (2) (RWF 15 500/US$19) Note: An additional total of RWF 2000/US$2 per household is spent on carrier bags for each of the above.
End of study	LPG stove (PEN 150/US$46)+1 full tank of LPG (PEN 95/US$29)+valve and hose (PEN 13/US$4)+pressure cooker (PEN 120/US$36)	Coupon worth GTQ 500/US$67, to be saved or redeemed for items from a catalogue Coupons exchangeable for specified household items at each visit or for a stove at the end with all coupons (GTQ 2,000/US$268).	Two-burner LPG stove (INR 2500/US$35) Security deposit for two 14.2 kg cylinders (INR 2900/US$41) Security deposit for pressure regulator (INR 150/US$2) LPG stove stand, rubber tube, installation and demonstration charges, administrative charges for documentation, domestic gas customer card (INR 2716/US$39) 18 LPG refills (INR 15 300/US$217) Lighter (INR 135/US$2)	Choose 1: LPG stove +1 full cylinder +4 LPG refills; A solar home system; or cash deposit (RWF 178 616/US$215)
Total cost of compensation package per control household	PEN 463/US$141, plus PEN 35/US$11 in HHs with older adult women	GTQ 2000/US$268	INR 25 601/US$363	RWF 259 624/US$313

*Exchange rates: US$1=3.3 Peruvian nuevo soles (PEN; Peru), 7.7 Guatemalan quetzales (GTQ; Guatemala), 70.44 Indian rupees (INR; India), 830 Rwandan francs (RWF; Rwanda).

†Compensation provided to all participants (intervention and control) in India.

In Peru and Rwanda, for example, gift items will be provided to control participants at designated points throughout their participation in the trial. These items were mentioned during the formative research: sweaters and hats in the case of Peru and *kitenge* cloth in the case of Rwanda. At baseline, all Rwandan participants will receive a table (which serves as an LPG stove base for intervention households). At the conclusion of HAPIN participation, control households in Peru will receive an LPG stove and cooking package (stove, one cylinder of LPG and pressure cooker). Rwandan controls will be able to choose from several options at the end of the study: an LPG package, a solar kit, or a cash equivalent.

The Guatemala IRC decided on a different approach: control participants would be able to choose from a catalogue of items of varying value, with included items selected by working group members. Control households will receive a total of four coupons worth GTQ 500 (US$67) each, which they can use to purchase items at the time of receipt or apply towards the purchase of a larger item at the study’s end. The wait would increase in proportion to an item’s value: higher-priced items (LPG stove, refrigerator, dining table and chairs, clothes wardrobe) would require waiting until the end of the trial. A shorter wait would be required for smaller items (eg, clothes iron, set of dishes, blender).

In India, formative research suggested that cash compensation—equivalent to the amount spent on stoves and LPG fuel for an intervention household—might be appropriate for controls. India has previously used conditional cash transfers successfully to motivate desired behaviours such as giving birth in health facilities.^6^ This cash-based compensation plan, however, was rejected by the Sri Ramachandra Institute of Higher Education and Research ethics board. The board stipulated that the India team must not provide monetary compensation to either study arm except to reimburse transportation costs and lost wages, and directed that at the end of the trial, the India IRC research team must provide controls with exactly the same package that intervention households receive. As this advice generally aligned with the multi-country guidance, it was decided that control households in India would be given an LPG stove identical to the one received by intervention households, plus credit towards an equal amount of LPG. Institutional review boards and ethics boards in the other three IRCs allowed plans as proposed.

## Lessons learned

Initially, HAPIN planned to compensate control participants at trial’s end with a package identical to that received by intervention households: an LPG stove plus 18 months of fuel. It soon became clear that this approach presented a variety of problems, from potential to introduce differential bias into the study to concerns about the fairness of asking control households to undergo multiple study procedures over an 18-month period without ongoing compensation. After integrating input from local staff, formative research, study community members, ethics committees, and experts, the plan that emerged instead consisted of a set of compensation packages based on uniform guiding principles and adapted to the different contexts of the four IRCs. These were implemented in April 2018 as enrolment began. As the trial progresses, HAPIN investigators are closely monitoring control compensation packages to ensure they are meeting the original goals (ie, to maximise engagement and minimise differential attrition in the control arm).

Different models for compensating research participants have been suggested in the literature.^7–9^ According to the *free market* model, participants would be paid competitive rates based on the principles of supply and demand (eg, adjusted to meet recruitment goals). Under *wage-payment*, payment would align with local rates for unskilled labour and would be provided based on the time participants devote to research activities. The *reimbursement* model suggests reimbursement of actual expenses (eg, transport and lost wages). *Appreciation* models suggest that research participation is less a form of labour than a type of altruistic engagement, and that compensation therefore should not be based on market rates but rather seen as an expression of gratitude for participating. These models are not mutually exclusive, and scholars have suggested that ‘the best way of establishing an appropriate level of payment may be to use some combination of these different approaches, depending on the type of research being conducted’.^7^

These models were never explicitly discussed during development of the HAPIN control compensation strategies. Examination after the fact, however, suggests that the strategies combined elements of several models. The predominant model combined ‘free market’ principles (because compensation levels were designed to be high enough to encourage recruitment and retention) with ‘appreciation’ (because in general the items provided—particularly the baby and household items—were explicitly framed as ‘gifts’ and thus distanced from the language of work and labour). India was the only IRC to employ the ‘reimbursement’ and ‘wage-payment’ models alongside the others, in its provision of travel and lost-wage stipends to participants.

Limited research exists on preferred compensation mechanisms in low-income and middle-income settings. Participants in a Kenya study preferred cash payments, due to their flexibility.^5^ However, these participants distinguished between reimbursements for direct costs (eg, transportation and lost wages)—which were highly preferred in cash—and tokens of appreciation, for which goods were preferred. An important consideration here may be that biomass-using households are on the lower end of the socioeconomic spectrum. One common argument in the literature suggests that financial remuneration is more attractive to economically disadvantaged populations and might lead to distorted perceptions of risk.^10^ Others, however, argue that removing cash from the menu of options for the socioeconomically disadvantaged is a form of paternalism that limits the options of research participants rather than protecting them^8^ and could actually disproportionately exclude poorer participants.^2^

No other site besides India included cash reimbursements (for travel or wages) as compensation. Cash as a form of appreciative compensation, meanwhile, appears only in Rwanda, where a cash payment is among the options that may be chosen by control households at the conclusion of the trial. The other IRCs all decided on goods-based compensation, though some (Guatemala and Rwanda) incorporated elements of flexibility and choice into their packages.

Though not explicitly outlined in the multi-country guidance (table 1), equity between control and intervention arms came up repeatedly in community and ethics board discussions. The clearest example was in India, where an ethics board mandated that control households receive a compensation package identical to the intervention one. This makes India the only setting in which control and intervention packages are monetarily equal, each valued at US$383 (see online Supplementary tables 1 and 3). In the other settings, the market value of the intervention package remained greater than of the control package, sometimes significantly so. In Guatemala’s case, the intervention cost is US$1414 per household versus US$268 in compensation for each control household. These differences reflect the cost of achieving the aims of the intervention in each locale. Each IRC designed their intervention according to a primary objective: to provide an LPG stove, fuel and any needed additional supplies that would enable households to complete all their cooking tasks exclusively and safely with this stove and fuel. For example, in Guatemala, a griddle is needed to cook tortillas, a staple food. The Guatemalan IRC chose a three-burner stove incorporating a griddle as their intervention stove, which raised costs here. Although we believe that equity between intervention and control households in HAPIN is best calibrated to the burden on study participation rather than the financial expense of delivering the intervention, these details highlight the complexity of deciding what is ‘fair’ when interventions are seen as having actual market value.

10.1136/bmjgh-2019-001567.supp1 Supplementary data

In Rwanda, a key challenge uncovered during formative research was that few rural households had prior experience with LPG. Providing an LPG stove at trial’s end, therefore, was not viewed as favourably in Rwanda as in India, where the nation-wide Pradhan Mantri Ujjwala Yojana campaign has increased LPG awareness and accessibility, even in rural areas.^11^ LPG access has also been increasing rapidly in Peru, partly due to a nation-wide government subsidy programme that provides discounted LPG to poor, rural families.^12^ In Rwanda, it was important to offer other compensation items valued in this community: in this case, a choice between an LPG package, a solar kit and/or a cash deposit equivalent in value to the two other options. In Guatemala, awareness of LPG was higher but formative research participants worried they would likely not be able to afford to purchase LPG regularly post-trial. The Guatemala IRC decided to allow control group participants to choose their compensation, given that an LPG stove provided at the end might have little value to households who could not afford enough fuel to use it regularly.

The third criterion for control compensation was that strategies minimise the potential economic advantage created by the LPG stoves and fuel via reductions in purchased fuel costs and in time spent cooking and collecting fuel. In the end, however, HAPIN’s team concluded that the direction and strength of any potential economic effect associated with the provision of free LPG fuel to HAPIN households is not something that can be assumed a priori, but rather is a research question of its own to be investigated over the course of the trial. For example, many HAPIN participants typically collect biomass fuel themselves, drawing on abundant and accessible supplies of wood and/or animal dung. Receiving free LPG where biomass fuel is already free may confer no economic advantage that could improve health status. It is, likewise, not yet clear how much time savings will accrue from avoided fuel collection, nor how participants might spend any such extra time. As a result, plans are underway to track and evaluate the economic impact of the intervention during the HAPIN trial via dietary surveys, questionnaires and time use studies. While not available until the trial concludes, data on these outcomes will contribute to the scant extant literature on the effects of in-kind transfers on economic and health outcomes in low-income and middle-income countries (eg, Gentilini,^13^ Cunham^14^ and McIntosh^15^).

## Conclusions

This paper illustrates how formative research, guided by common principles, can be used to develop cross-cutting but locally appropriate strategies for compensating control group participants in a multi-country RCT. The diversity of compensation packages developed for HAPIN’s four countries suggests that one-size-fits-all approaches may not achieve their intended goals in multi-country trials. Agreeing on general principles as central guidance, however, can usefully inform local implementation of contextually appropriate control compensation packages in low- and middle-income settings.
