# Supplementary material for: Compensating control participants when the intervention is of significant value: experience in Guatemala, India, Peru and Rwanda
Source: BMJ Glob Health. 2019 Aug 21;4(4):e001567. doi: 10.1136/bmjgh-2019-001567 (PMC6730613; doi:10.1136/bmjgh-2019-001567)
Supplement: Supplementary data [file bmjgh-2019-001567supp001.pdf]

Supplementary Material

Supplementary Table 1: HAPIN Intervention packages and cost per household (intervention households only)

	Peru	Guatemala	India	Rwanda
**Stove**	Three-burner LPG stove (PEN* 150/ USD 46)	Custom-designed 3-burner LPG stove with built-in *comal* (griddle for cooking tortillas) (GTQ* 1340/USD 174)	Two-burner LPG stove (INR* 2500/ USD 35)	Three-burner LPG stove (RWF 32,000 /USD 39)
**Cylinders and LPG setup materials**	Two 10 kg LPG cylinders (PEN 120/ USD 36)<br><br>Valve and hose (PEN 12/ USD 4)	Two 11.3kg cylinders (GTQ 710/ USD 92)<br><br>LPG kit: 2 POL valves, 3m hose, regulator, copper connection tubes, 2 clamps = (GTQ316/ USD 41).	Security deposit for two 14.2 Kg cylinders (INR 2900 / USD 41.12)<br><br>Security deposit for pressure regulator (INR 150 / USD 2.13)<br><br>LPG stove stand, rubber tube, installation & demonstration charges, administrative charges for documentation, domestic gas (DGCC) customer card (INR 2716/ USD 38.55)<br><br>Lighter (INR 135 / USD 1.91)	Two 15 Kg cylinders (RWF 35,000 /USD 42.2 each, empty)<br><br>LPG Kit: Two click-on adaptors, two high pressure hoses, one regulator and hose nozzle, one manual change over valve (€32), one low pressure hose and clips (RWF 2,750). (Total USD 43)
**Fuel****	Approximately 2 LPG refills/mo. (20 kg) for 18 months (PEN 1,278/ USD 387)	Approximately 4 LPG refills/mo. (45.4 kg) for 18 months (GTQ 8136/ USD 1057)	Approximately 1 LPG refill/mo. (14.2 kg) for 18 months (INR 15,300 / USD 217)	Approximately 1.25 LPG refills/mo. (19Kg) for 18 months (RWF 372,670 /USD 449)
**Additional cooking-related items**		Set of pots and pans (GTQ 220/ USD 29)<br><br>Calendar with cooking tips (GTQ 160/ USD 21)		Table (RWF 8,000 /USD 9.60), Roasting appliance (RWF 7,000 /USD 8), 4 cooking Posters (RWF 5,146 /USD 6)
**Material cost of intervention**	PEN 1560/ USD 473	GTQ 10,882/ USD 1414	INR 23701/ USD 336	RWF 529,872 /USD 638
**Remaining with the household post-trial**	Stove and one cylinder (PEN 210/ USD 64)	Stove, one cylinder, LPG Kit, set of pots and pans and calendar (GTQ 2395/ USD 311)	Stove, two cylinders, pressure regulator, rubber tube, stove stand, lighter, DGCC book (INR 8194 / USD 116)	Stove, one cylinder, one hose, clip and one regulator, table, roasting appliance and 4 posters. (RWF 99,896) / USD 120)
**Additional compensation**	Gift bag at birth visit with diaper bag, baby clothes, baby blanket (PEN 35/ USD 11)	--	Mock randomization lunchbox (INR 50/ < USD 1) †<br><br>Newborn gift box with baby clothes, baby	--

**Supplementary Table 1: HAPIN Intervention packages and cost per household (intervention households only)**				
	**Peru**	**Guatemala**	**India**	**Rwanda**
			towel (INR 200 / USD 3) †<br><br>Travel/food allowance for clinic assessments (INR 200/ USD 3 x 3 visits = INR 600/ USD 9) †<br><br>Time compensation for each home assessment visit (INR 150/ USD 2 x 7 visits = INR 1050/ USD 14) †	
**Total cost per household**	PEN 1595/ USD 484	GTQ 10,882/ USD 1414	INR 25601 / USD 363	RWF 529,872/ USD 638

* Exchange rates: US Dollars (USD) $1 = 3.3 Peruvian nuevo soles (PEN), 7.7 Guatemalan quetzales (GTQ), 70.44 Indian rupees (INR), 830 Rwandan francs (RWF)

** Note, households are provided as much fuel as needed to fulfill all household cooking tasks. Differing amounts across IRCs reflect differences in cooking habits.

† Compensation provided to all participants (intervention and control) in India
